# Optimization of ultrasonic extraction processes, structural characteristics and potential antipyretic mechanism of a glucan from *Tetrastigma hemsleyanum Diels*

**DOI:** 10.1016/j.ultsonch.2025.107456

**Published:** 2025-07-06

**Authors:** Zhongpeng Ding, Ningchen Zhang, Meihong Ding, Shiyi Zhang, Xiaowen Yao, Yu Xia, Senlin Shi

**Affiliations:** College of Pharmaceutical Sciences, Zhejiang Chinese Medical University, Hangzhou 311400, China

**Keywords:** *Tetrastigma hemsleyanum Diels*, Polysaccharide, Antipyretic, Structural characteristic

## Abstract

*Tetrastigma hemsleyanum Diels*, a rare Chinese endemic medicinal plant, contains bioactive polysaccharides with therapeutic potential. This study systematically optimized ultrasound-assisted polysaccharide extraction through artificial neural network modeling, followed by comprehensive structural characterization of the purified polysaccharides. We further investigated the antipyretic efficacy of polysaccharides and revealed preliminary insights into their molecular mechanisms. A polysaccharide designated ADHP1 was isolated from Tetrastigma hemsleyanum Diels. Structural characterization identified ADHP1 as a glucosan with a predominantly (1 → 4)-α-linked Glcp backbone, featuring C-6 branching via T-α-linked Manp residues, and exhibiting a molecular weight of 156.42 kDa. In murine febrile models, ADHP1 demonstrated significant antipyretic activity by suppressing the overproduction of inflammatory cytokines (TNF-α, IL-1β) and mediators (cAMP, PGE_2_). Integrative computational analysis combining GEO data mining, PPI networks, and molecular docking revealed strong binding affinities between ADHP1 and targets CTSG, LTF, MPO, and PRTN3. This evidence establishes ADHP1 as a botanically-derived antipyretic agent, demonstrating dual prophylactic and therapeutic efficacy in febrile management through targeted immunometabolic modulation.

## Highlights

• Box-Behnken model, BP and GA-ACO-BP artificial neural network models were used to optimize the ultrasonic-assisted extraction process of ADHP1 to obtain the best extraction process.
• The composition and structure of ADHP1 were elucidated.
• Treatment of fever with ADHP1 shows initial therapeutic promise
• ADHP1 is the potential ingredient to develop the functional drug containing plant-derived THD.

## Graphical abstract

**Figure:**
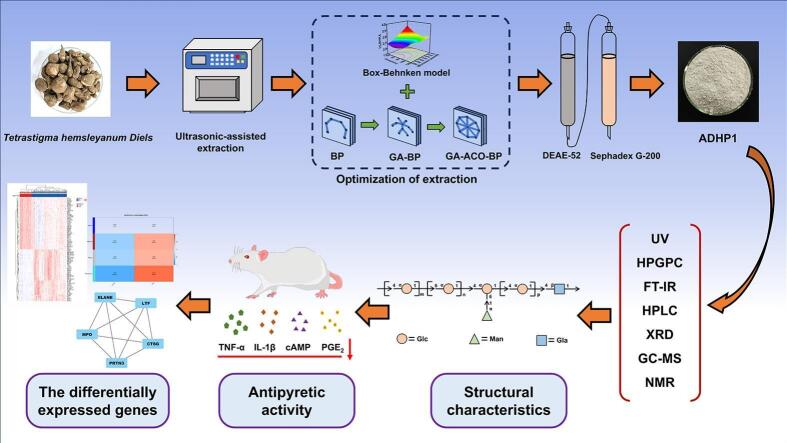

## 1 Introduction

The *Tetrastigma hemsleyanum Diels* (THD) is a unique and precious herbal medicine in China, which has been widely used for more than 1000 years [1]. According to the “Compendium of Materia Medica”, THD was historically used for treating fever, febrile convulsion in children, dysentery, and hepatitis [2]. Moreover, modern studies have showed THD has a variety of medicinal properties, e.g., anticancer, antioxidant, antiviral, analgesia, and hepatoprotective [[3], [4], [5]]. In addition to small molecule compounds, e.g., flavonoids, phenolic acids and steroid alkaloids, the polysaccharide has been proved to be an important active component in THD [[6], [7], [8], [9]]. However, few studies related to the pharmacological effects of the THD polysaccharides were reported, and their specific pharmacological characteristics are still unclear.

It is well-known that the structural characteristics of polysaccharides determine their bioactive properties [[10], [11], [12]]. The biological activity and therapeutic efficacy of polysaccharides are determined by multiple structures, including molecular weight, three-dimensional conformation, length of the chain, branching patterns, and specific sugar groups [13]. Although preliminary investigations have characterized certain structural features of THD polysaccharides, current analytical data remain insufficient for comprehensive elucidation of these polysaccharide architectures. This knowledge gap underscores the imperative for systematic structural characterization coupled with functional analyses to establish definitive structure–activity relationships governing THD polysaccharide pharmacology.

This study posits that the antipyretic efficacy of THD polysaccharides is governed by their distinct structural architecture, particularly the molecular features of the purified neutral polysaccharide ADHP1. We hypothesize that systematic optimization of ultrasonic extraction parameters and rigorous structural characterization will reveal critical structure–activity relationships underlying ADHP1′s bioactivity. Furthermore, we propose that ADHP1 alleviates fever through targeted interactions with specific molecular receptors or pathways, as evidenced by integrative pharmacological validation and computational analyses of protein-receptor dynamics.

To test this hypothesis, this study implemented a dual-model optimization framework combining Box-Behnken with neural network to systematically optimize ultrasonic-assisted extraction parameters, employing THD polysaccharide yield as the response variable. Subsequently, the crude extract underwent were purified by DEAE ion-exchange chromatography to obtain the neutral polysaccharide ADHP1. The molecular architecture of ADHP1 was systematically elucidated through a multi-technique analytical platform integrating spectroscopy with chromatography. Then, the protective and alleviating effects of ADHP1 were explored in the mice model of fever. Furthermore, GEO (Gene Expression Omnibus) data mining, PPI (Protein-Protein Interaction Network) and molecular docking were performed to elucidate possible receptors for the antipyretic effects of ADHP1. Our findings provide important insights into the application of THD polysaccharides, the relationship between ADHP1 structure and fever, and potential therapeutic strategies.

## 2 Material and methods

### 2.1 Material and chemicals

The *Tetrastigma hemsleyanum Diels* were obtained from medicinal botanical garden of Zhejiang Chinese Medical University and identified by Prof. Senlin Shi. Aspirin tablets were bought from Shenwei Co., Ltd (Cat. No. 24040811). Dry yeasts were bought from Angelyeast Co., Ltd (Cat. No. CY20240308). Mouse TNF-α ELISA Kit, Mouse IL-1β ELISA Kit, Mouse cAMP ELISA Kit and Mouse PEG_2_ ELISA Kit were bought from Jiangsu Meimian Co., Ltd (Cat. No. 202501).

### 2.2 Extraction and determination of polysaccharide

Polysaccharide extraction was performed via an ultrasonic-assisted (Ultrasonic extraction instrument, KQ-500DE, KSUI Inc., China) method wherein 65-mesh THD powder was combined with ultrapure water in a conical flask prior to sonication treatment [14]. Following ethanol-induced precipitation (80 % v/v) of polysaccharides from the extract, the resultant precipitate was isolated through centrifugation (8,000 × g, 15 min) and subsequently lyophilized (Freeze dryer, AD2.0 EL, SP Scientific Inc., USA) to constant mass. Polysaccharide content was quantified using the phenol–sulfuric acid method, and ADHP yield was calculated with Eq. (1).

(1) $$\mathrm{yield}\left(\%\right)=\frac{\mathrm{Polysaccharide}\;concentration\;\left(\frac{g}{\mathrm{mL}}\right)\times Volume\;\left(mL\right)}{\mathrm{Tetrastigma}\;hemsleyanum\;Diels\;weight\;(g)}\times 100\%$$

### 2.3 Optimization of extraction process

A multifactorial optimization framework integrating artificial neural network (ANN) modeling, Box-Behnken design (BBD) response surface methodology, and single-factor parameter screening was implemented to maximize polysaccharide yield. Critical process parameters (liquid–solid ratio [15–30 mL/g], extraction temperature [40–100 °C], ultrasound intensity [250–550 W], and duration [30–120 min]) were systematically evaluated through four-level factorial experimentation as detailed in Table 1. Predictive model performance was comparatively assessed using goodness-of-fit metrics (R^2^, MAE and RMSE) and experimental validation.

**Table 1:** Variables and their levels.

Factors	Level			
Liquid-solid ratio	15	20	25	30
Extraction temperature (°C)	40	60	80	100
Ultrasonic power (W)	250	350	450	550
Extraction time (min)	30	60	90	120

#### 2.3.1 Single-factor experimental design

Univariate parameter optimization was systematically conducted through iterative single-factor variation (four equidistant levels per parameter) to establish operational boundaries for subsequent BBD response surface modeling.

#### 2.3.2 BBD response surface methodology

Building upon empirical parameter boundaries established in preliminary univariate analyses, a four-factor, three-level Box-Behnken model (29 experimental runs with 5 central points) was implemented via Design-Expert 12.0 (Stat-Ease Inc., USA) to establish optimal process conditions (Table 2). Process optimization was achieved through response surface methodology incorporating ANOVA-validated quadratic regression models, with polysaccharide yield (%) as the objective function.

**Table 2:** Comparison of true and predicted values from 29 extraction experiments.

Run	Liquid-to-solid ratio (mL/g)	Ultrasonic power (W)	Extraction temperature (°C)	Time (min)	Yield (%)	Box-Behnken Predicted	BP Predicted	GA-ACO-BP Predicted
1	20	90	60	300	13.70	14.96	13.02	13.36
2	25	100	75	250	16.32	16.48	16.36	16.64
3	25	90	75	300	15.15	16.12	15.97	16.56
4	25	100	90	300	15.71	16.47	15.73	15.71
5	25	90	90	250	16.41	15.92	16.41	16.41
6	25	90	75	300	14.76	16.12	15.97	16.56
7	25	90	60	350	13.59	14.31	14.13	13.59
8	20	90	75	250	17.05	16.03	16.16	15.73
9	20	90	75	350	16.72	16.1	16.44	16.72
10	25	80	75	250	8.08	9.23	8.12	8.08
11	25	90	75	300	16.85	16.12	15.97	16.56
12	30	90	60	300	15.96	15.32	15.87	15.96
13	25	90	75	300	18.07	16.12	15.97	16.56
14	30	90	90	300	17.66	17.19	17.59	17.66
15	20	90	90	300	14.94	16.36	15.73	14.94
16	30	80	75	300	8.85	9.90	8.82	8.85
17	20	100	75	300	16.22	15.4	16.36	15.67
18	25	90	90	350	17.11	16.72	17.54	15.94
19	30	90	75	350	16.65	16.66	17.25	16.65
20	25	100	75	350	15.73	15.37	16.64	15.73
21	25	100	60	300	14.75	14.57	16.41	14.75
22	20	80	75	300	10.87	10.66	9.80	10.87
23	30	90	75	250	17.06	16.66	16.25	16.74
24	25	90	60	250	14.44	15.05	14.79	14.41
25	30	100	75	300	16.91	17.36	15.91	17.21
26	25	80	90	300	10.93	10.10	11.41	11.10
27	25	80	60	300	10.51	8.73	10.37	10.34
28	25	90	75	300	15.76	16.12	15.97	16.56
29	25	80	75	350	9.78	10.41	10.24	9.89

#### 2.3.3 BP and GA-ACO-BP artificial neural networks

Building upon the BBD-optimized parameter space, a comparative neural architecture was implemented using MATLAB R2020b (MathWorks Inc., USA) comprising both standard BP and GA-ACO-BP architectures [15,16]. The network topology featured four input neurons corresponding to process variables (liquid–solid ratio, extraction temperature, ultrasonic power, time) and a single output neuron encoding polysaccharide yield. The experimental dataset (n = 29) was partitioned into stratified training (n = 21) and validation (n = 8) subsets. Hidden layer dimensionality (6–12 neurons) was optimized via golden-section search minimizing mean squared error during supervised learning. Model fidelity was quantified through tripartite metrics: R^2^, MAE, and RMSE, calculated per Eqs. (2), (3), (4) with statistical significance verified via paired *t*-test.

(2) $$R^{2}=ESS/TSS\;=1-RSS/TSS=1-{\sum_{i=1}^{n}\left(Y_{i,a}-{\tilde{Y}}_{i,p}\right)}^{2}/{\sum_{i=1}^{n}\left({\overset{¯}{Y}}_{a}-{\tilde{Y}}_{i,p}\right)}^{2},\;\;k=1,\;2$$

(3) $$\mathrm{MAE}=\left(1/n\right)\sum_{i=1}^{n}\left|Y_{i,a}-{\tilde{Y}}_{i,p}\right|,\;\;k=1,\;2$$

(4) $$\mathrm{RMSE}=\sqrt{\left(1/n\right)\sum_{i=1}^{n}{\left(Y_{i,a}-{\tilde{Y}}_{i,p}\right)}^{2}},\;\;k=1,\;2$$

Among them, the ${\tilde{Y}}_{i,p}$ is each model forecast, $Y_{i,a}$ is the actual value, ${\overset{¯}{Y}}_{a}$ is the average of the data set.

### 2.4 Isolation and purification of polysaccharide

Protein removal was achieved through Sevage deproteinization [17]. The crude polysaccharide extract underwent sequential fractionation using a DEAE-52 anion-exchange chromatography system with a 0–0.8 M NaCl gradient elution. Fractions containing polysaccharides were pooled, lyophilized, and quantified via phenol–sulfuric acid assay. Subsequent purification employed Sephadex G-200 gel filtration chromatography using ultrapure water as mobile phase. The target fraction was collected, freeze-dried, and designated as ADHP1 [18].

### 2.5 Characterization of polysaccharide

#### 2.5.1 Content measurement of protein and uronic acid

The ADHP1 fraction underwent comprehensive spectroscopic characterization through UV–visible spectrophotometry (UV, 2600i, SHIMADZU Inc., Japan) (200–800 nm scan range, 1 nm resolution). Protein content of ADHP1 was quantified via coomassie brilliant blue method with bovine serum albumin calibration, while uronic acid content was determined by *meta*-hydroxydiphenyl colorimetric analysis following 30 min sulfamic acid-catalyzed reaction at 80 °C, standardized against D-glucuronic acid. All spectrophotometric measurements were performed in triplicate with appropriate solvent blanks.

#### 2.5.2 Measurement of molecular weight

Molecular weight distribution and homogeneity of ADHP1 were analyzed by HPGPC (GPC 1515, Waters Inc., USA). The separation protocol followed established chromatographic parameters [19]. Calibration was performed with characterized dextran molecular weight standards to generate a retention correlation curve.

#### 2.5.3 Congo red assay

The conformational analysis of ADHP1 was conducted via a Congo red (CR) binding assay. An ADHP1 aqueous solution (1.0 mL, 2.50 mg mL^−1^) was mixed with a CR solution (1.0 mL, 80.0 μmol/L) and incubated at 25.0 ± 0.2 °C for 30 min to equilibrate. NaOH (1.00 mol/L) was then introduced into the mixture to attain final NaOH concentrations of 0–0.50 mol/L. UV (scan range: 200–800 nm) was employed for spectral characterization. Control experiments using CR-only solutions verified baseline stability. Characteristic bathochromic shifts in the spectra confirmed the triple-helix conformation [20].

#### 2.5.4 FTIR spectral analysis

The dried ADHP1 powder was homogeneously blended with KBr powder at a 1:10 (w/w) ratio and subsequently compressed into translucent sheet. FTIR spectroscopy (Nicolet IS50, Thermo Fisher Inc., USA) was then employed to acquire spectral data across the 500–4000 cm^−1^ range.

#### 2.5.5 Monosaccharide composition

Monosaccharide composition analysis was conducted via acid hydrolysis in 2 M TFA at 110 °C for 4 h, followed by PMP derivatization under alkaline conditions (0.3 M NaOH, 30 min) and subsequent neutralization with 0.3 M HCl [21]. Separation was performed on a HPLC system (Agilent 1200, Agilent Inc., USA) equipped with a C18 column. Component identification utilized retention time matching against certified monosaccharide standards, with quantitative analysis achieved through external calibration based on integrated peak areas.

#### 2.5.6 XRD analysis

Crystallographic characterization of ADHP1 was performed using XRD (XRD-6100, SHIMADZU Inc., Japan) analysis with Cu Kα radiation (λ = 1.5406 Å) at 40 kV tube voltage. Diffractograms were acquired across a 2θ angular range of 5°–80° with a scanning rate of 2°/min.

#### 2.5.7 Methylation analysis

ADHP1 (5 mg) was sequentially methylated, hydrolyzed, reduced, and acetylated to generate partially methylated alditol acetates (PMAAs). PMAAs were analyzed by GC–MS (7890B, Agilent Inc., USA) following established protocols [20].

#### 2.5.8 Nuclear magnetic resonance

For structural elucidation, ADHP1 (20.0 mg) was deuterated in high-purity D_2_O (1.0 mL) via three lyophilization cycles. One-dimensional ^1^H and ^13^C spectra were recorded using presaturation-based solvent suppression. Two-dimensional analyses comprised the following experiments: COSY, HSQC, and NOESY, all acquired with 2-s relaxation delays (NMR, AVANCE Ⅲ 600 MHz, BRUKER Inc., Germany).

### 2.6 In vivo antipyretic activity

#### 2.6.1 Animals and ethics statement

ICR mice (Half male and half female, mean body weight 20 ± 2 g) were sourced from the Experimental Animal Center of Zhejiang Chinese Medical University (License No. SCXK(Zhe)2022-0005). The research subjects were housed in a controlled environment maintaining 21 ± 1 °C ambient temperatures, 40–70 % humidity, and standardized 12-h photoperiod cycles. All in vivo studies were approved by the Institutional Animal Care Committee at Zhejiang Chinese Medical University (approval number: IACUC-20240122-11), and all procedures were conducted in strict accordance with the National Research Council's Guide for the Care and Use of Laboratory Animals.

#### 2.6.2 Experimental design

During the 3-day pre-modeling phase, rectal temperatures were measured bidaily (08:00 and 20:00) using a calibrated digital thermometer (DT008, BRK Inc., China). The mean values from 3-day measurements served as individual basal body temperature references. Mice exhibiting either a basal temperature exceeding 38.0°C or diurnal fluctuations surpassing 0.5 °C were excluded from subsequent experimentation.

Mice were randomly divided into six groups (n = 8) based on rectal temperature: (1) control, (2) model, (3) aspirin-treated positive control (0.2 g/kg), and (4–6) ADHP1-treated groups receiving 400, 200, or 100 mg/kg doses. Except for the control group, the fever model was established by subcutaneous injection of 20 % dry yeast suspension (10 ml/kg) in other groups [22]. After 6 h, the rectal temperature increased by 0.8 °C or more was considered to be successful. Then, the mice were given orally by gavage immediately, and the rectal temperature changes were measured within 6 h after administration.

Except for the control group, the fever model was achieved in other groups through subcutaneous administration of 20 % (w/v) dry yeast suspension (10 mL/kg). Successful model establishment was confirmed by rectal thermometry 6 h post-injection, with inclusion criteria requiring ≥0.8 °C elevation from baseline. Immediately following model validation, therapeutic interventions were administered via oral gavage. Post-treatment rectal thermometry was quantified at 60 min intervals for 6 h using digital thermometer.

After the last temperature measurement, mice were anesthetized, and serum was collected for TNF-α and IL-1β assays. Subsequently, the whole brain was quickly removed, the blood was removed by saline flushing, and the hypothalamus was isolated and stored in a refrigerator at −80 °C for cAMP and PGE_2_ detection [23].

Following terminal temperature monitoring, mice underwent Zoletil 50 anesthesia (50 mg/kg, *i.p*.). Serum samples were obtained via cardiac puncture and centrifuged at 4 °C (3000*g*, 15 min) for subsequent TNF-α and IL-1β quantification using ELISA kits. Subsequently, the whole brain was quickly removed, rinsed with saline to remove surface blood, and the hypothalamus was isolated and stored at −80 °C for cAMP and PGE_2_ quantification using ELISA kits.

### 2.7 GEO data mining

The Gene Expression Omnibus (GEO) repository was utilized to acquire datasets GSE185576 and GSE101702 through their respective accession identifiers [24]. The GSE185576 and GSE101702 datasets provided transcriptomic profiles of 311 peripheral blood samples, including 76 healthy controls and 234 febrile patients. Probes were annotated using the matched GPL21185 platform file. Bioinformatic interrogation of the GSE185576 and GSE101702 datasets employed a Student's *t*-test framework for differential expression screening, with statistically significant differentially expressed genes (DEGs) defined as those exhibiting |log_2_FC| > 1 and *p* < 0.05. To further explore gene interactions, Weighted Gene Co-expression Network Analysis (WGCNA) was applied to both datasets, enabling the construction of co-expression networks and module detection. Finally, protein–protein interaction (PPI) network analysis facilitated the screening of central hub genes.

### 2.8 Molecular docking of ADHP1

Building upon DEG analysis findings, molecular docking simulations of ADHP1 were conducted to elucidate its potential antipyretic mechanisms. The three-dimensional crystal structure of the target protein was acquired from the RCSB Protein Data Bank. Molecular modeling of ADHP1 was initiated by constructing its three-dimensional conformation in Chem3D (CambridgeSoft Inc., USA). Quantum chemical optimization involving geometry refinement and partial charge derivation was executed through MOPAC employing the PM3 semi-empirical framework. Subsequent ligand preparation using AutoDock Tools (Scripps Research Institute, USA) generated docking-ready pdbqt coordinate files through systematic hydrogen addition and torsional parameterization [25].

### 2.9 Statistical analysis

Quantitative data are presented as mean ± SD derived from three independent experimental replicates. Statistical evaluations were conducted using GraphPad Prism 10 (GraphPad Software Inc., USA), with parametric one-way ANOVA employed to assess intergroup differences. A probability threshold of *p* < 0.05 defined statistical significance.

## 3 Results

### 3.1 Extraction optimization

#### 3.1.1 Single-factor experimental design

**Fig. 1:**
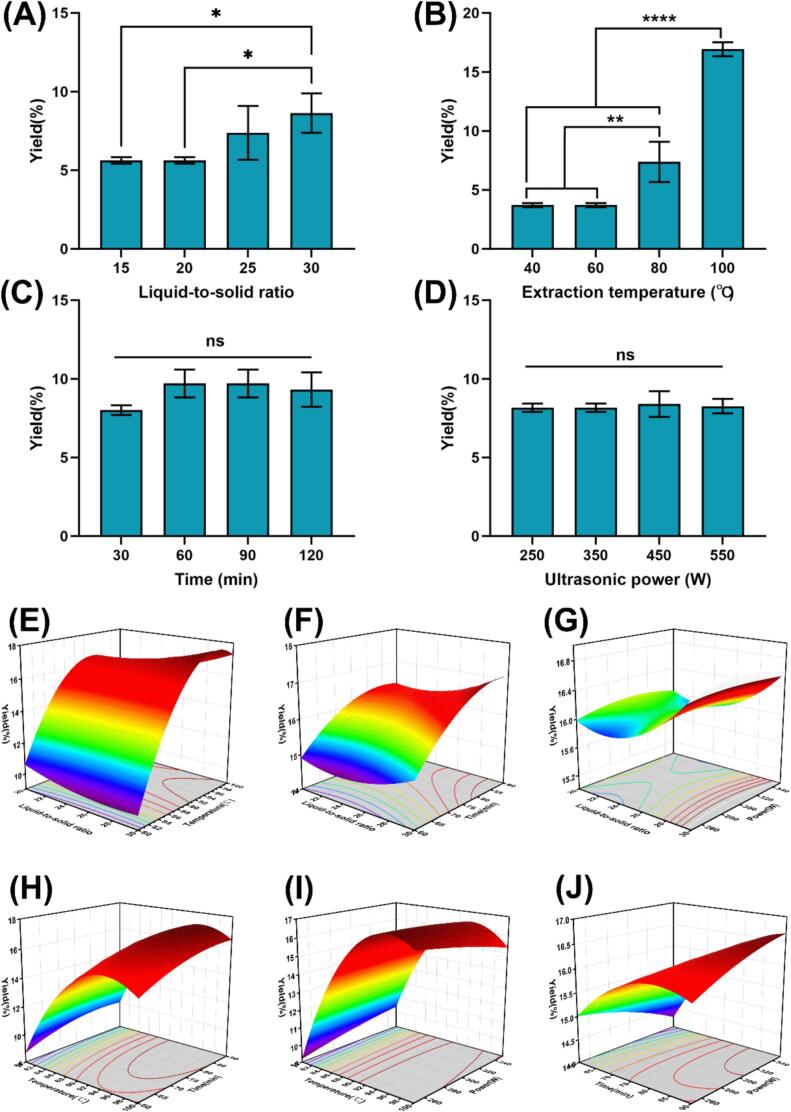
Effects of operational parameters on THD polysaccharide yield. (A) Liquid-to-solid ratio, (B) extraction temperature, (C) extraction time, (D) ultrasonic power, (E) liquid-to-solid ratio × temperature interaction, (F) liquid-to-solid ratio × time interaction, (G) liquid-to-solid ratio × ultrasonic power interaction, (H) temperature × time interaction, (I) temperature × ultrasonic power interaction, (J) time × ultrasonic power interaction. Statistical significance was determined by one-way ANOVA (*p < 0.05, **p < 0.01, ***p < 0.001, ****p < 0.0001).

Fig. 1(A–D) illustrates the effects of various parameters on THD crude polysaccharide production yield. While temporal duration and ultrasonic power exerted no significant impact on polysaccharide yield, the remaining variables demonstrated a positive correlation with increased production. Three critical levels for the Box-Behnken design were determined by selecting the peak response value and its two adjacent points: liquid-to-solid ratio (20:1, 25:1, 30:1 mL/g), extraction time (60, 75, 90 min), and temperature (80, 90, 100 °C). The THD crude polysaccharide yield plateaued beyond 250 W, presumably attributable to complete extraction at this power. So, 250, 300, and 350 W were selected as the representative input parameters for the Box-Behnken model design.

#### 3.1.2 Box-Behnken model analysis

The Box-Behnken model optimized the THD crude polysaccharide extraction process through 29 experimental trials. Table 2 summarizes the experimental and predicted polysaccharide yields, with actual values spanning 8.08 % to 18.07 %, while the data conformed to the empirical relationship described by Eq. (5):

(5) $$\mathrm{Yield}=-259.61186-1.95775\times A+5.89544\times B+0.120847\times C+0.091979\times D+0.013581\times A\times B+0.001540\times A\times C-0.000072\times A\times D+0.0009\times B\times C-0.001143\times B\times D+0.000515\times C\times D+0.14024\times A^{2}-0.031412{\times B}^{2}-0.002268\times C^{2}-0.000043\times D^{2}$$

where A is the liquid-to-solid ratio, B is the extraction temperature, C is the extraction time and D is the ultrasonic power. Eq. (5) revealed an inverse correlation between liquid-to-solid ratio and yield, whereas extraction temperature, ultrasonic power, and duration exhibited proportional relationships with yield. The variables ranked by their influence magnitude on the composite score followed the hierarchy: B > A > C > D. The Box-Behnken model was statistically validated through analysis of variance (Table 3). Extraction temperature exhibited a highly significant effect on polysaccharide yield (*p*-value < 0.0001), with model adequacy confirmed by Model *p*-value = 0.0001 < 0.05 and Lack of Fit *p*-value = 0.619 > 0.05. The C.V. % = 8.66 indicated moderate experimental reproducibility. While the model demonstrated R^2^ = 0.896, suboptimal alignment between adjusted R^2^ (0.792) and predicted R^2^ (0.540) suggested limited predictive reliability. To elucidate variable interactions, three-dimensional response surface plots (Fig. 1E–J) were generated, mapping the relationships between the four parameters and polysaccharide yield. The Box-Behnken design predicted a maximum polysaccharide yield of 18.14 % under optimized conditions: 30:1 mL/g liquid-to-solid ratio, 340 W ultrasonic power, 95 °C extraction temperature, and 90 min duration.

**Table 3:** ANOVA results for the Box-Behnken experimental design.

Source	Sum of Squares	df	Mean Square	F-value	*p*-value	
Model	195.69	14	13.98	8.62	0.0001	significant
A	1.07	1	1.07	0.6589	0.4305	
B	111.82	1	111.82	68.91	< 0.0001	
C	8.04	1	8.04	4.96	0.0429	
D	0.0034	1	0.0034	0.0021	0.9643	
AB	1.84	1	1.84	1.14	0.3044	
AC	0.0534	1	0.0534	0.0329	0.8587	
AD	0.0013	1	0.0013	0.0008	0.9779	
BC	0.073	1	0.073	0.045	0.8351	
BD	1.31	1	1.31	0.8059	0.3845	
CD	0.5963	1	0.5963	0.3675	0.5541	
A^2^	0.7974	1	0.7974	0.4914	0.4948	
B^2^	64	1	64	39.45	< 0.0001	
C^2^	1.69	1	1.69	1.04	0.3248	
D^2^	0.0735	1	0.0735	0.0453	0.8346	
Residual	22.72	14	1.62			
Lack of Fit	15.48	10	1.55	0.8557	0.619	not significant
Pure Error	7.24	4	1.81			
Cor Total	218.41	28				
R^2^	0.896					
Adjusted R^2^	0.792					
Predicted R^2^	0.54					
C.V. %	8.66					

#### 3.1.3 BP and GA-ACO-BP neural network for extraction

Two artificial neural network models, BP and GA-ACO-BP, were developed to describe the nonlinear relationship between the four input variables and polysaccharide yield. Briefly, a BP neural network was constructed with an optimal number of hidden layer neurons of 4, a training number of 1000. Model parameters (weights and thresholds) were iteratively adjusted during training until achieving a convergence threshold of 0.00001. The optimization process terminated automatically upon reaching either the predefined iteration limit or a prediction error <0.00001 between calculated and experimental polysaccharide yields, with optimized parameters recorded in Table 2. Model parameters were iteratively optimized during training, achieving a minimum target error of 1 × 10^−5^. The iterative process terminated upon meeting either predefined epoch limits or error convergence criteria (output vs. true value <1 × 10^−5^), with final polysaccharide yield predictions documented in Table 2.

**Fig. 2:**
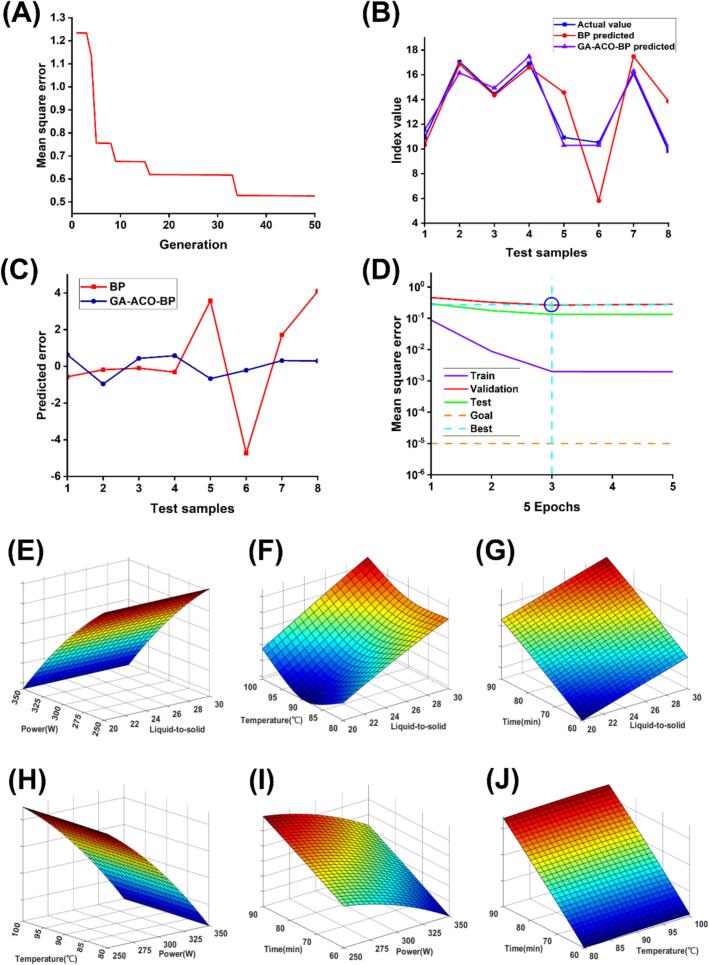
Performance evaluation of BP versus GA-ACO-BP neural networks. (A) MAE evolution across GA-ACO-BP training generations. (B) Predicted vs actual values comparison for BP and GA-ACO-BP networks. (C) Prediction error distribution between BP and GA-ACO-BP architectures. (D) Dataset-specific MSE performance of GA-ACO-BP networks. (E-J) Interaction effects between process parameters: (E) liquid-to-solid ratio and ultrasonic power, (F) liquid-to-solid ratio and extraction temperature, (G) liquid-to-solid ratio and time, (H) extraction temperature and ultrasonic power, (I) time and ultrasonic power, (J) time and extraction temperature.

The ant colony algorithm was hybridized with a genetic algorithm to establish an optimized GA-ACO-BP neural network. Fitness progression across generations was quantified through iterative calculations. Ant colony parameters included a pheromone-volatilization coefficient (ρ = 0.9), transition probability constant (α = 0.2), and pheromone initialization at unity concentration. Genetic operators optimized pheromone distributions, while ant colony dynamics governed pheromone updates during the iterative refinement phase. Network convergence was achieved at 34 iterations, evidenced by stabilized root mean square error (RMSE) trends (Fig. 2A). Final optimized weights and thresholds were transferred to the BP neural network, with training epochs and error thresholds mirrored from the original BP architecture. The GA-ACO-BP algorithm achieved a maximum polysaccharide yield of 19.15 % under optimized parameters: 30:1 mL/g liquid-to-solid ratio, 260 W ultrasonic power, 100 °C extraction temperature, and 90 min duration.

#### 3.1.4 Validation experiment

The predictive accuracy of the four models was evaluated using the R^2^, MAE, and RMSE. Model performance improved with higher R^2^ values accompanied by lower MAE and RMSE metrics [15]. The GA-ACO-BP model demonstrated the lowest prediction errors (MAE = 0.3673; RMSE = 0.6462), showing closest agreement with experimental data, whereas the BP model exhibited the largest deviations (MAE = 0.9533; RMSE = 1.3929) (Table 4). The GA-ACO-BP model demonstrated the highest predictive accuracy (R^2^ = 0.9446) among all tested models, confirming its superior performance. Comparative experimental validation revealed quantitative concordance with model predictions: polysaccharide yields reached 18.28 ± 0.40 % for the Box-Behnken model and 19.11 ± 0.33 % for the GA-ACO-BP model.

**Table 4:** Performance evaluation of Box-Behnken model versus neural network models (BP and GA-ACO-BP).

Model	R^2^	MAE	RMSE
Box-Behnken	0.8960	0.7520	0.8852
BP	0.7424	0.9533	1.3929
GA-ACO-BP	0.9446	0.3673	0.6462

In conclusion, the GA-ACO-BP model emerged as the superior predictive framework, yielding an extraction protocol that maximized polysaccharide production while exhibiting minimal deviation from experimental measurements. To visualize parameter-yield interdependencies, three-dimensional response surface plots (Fig. 2E–J) were derived from the GA-ACO-BP model, mapping mechanistic relationships between liquid-to-solid ratio, ultrasonic power, extraction temperature, time, and polysaccharide yield.

### 3.2 ADHP1 characterization

#### 3.2.1 Preparation of ADHP1

**Fig. 3:**
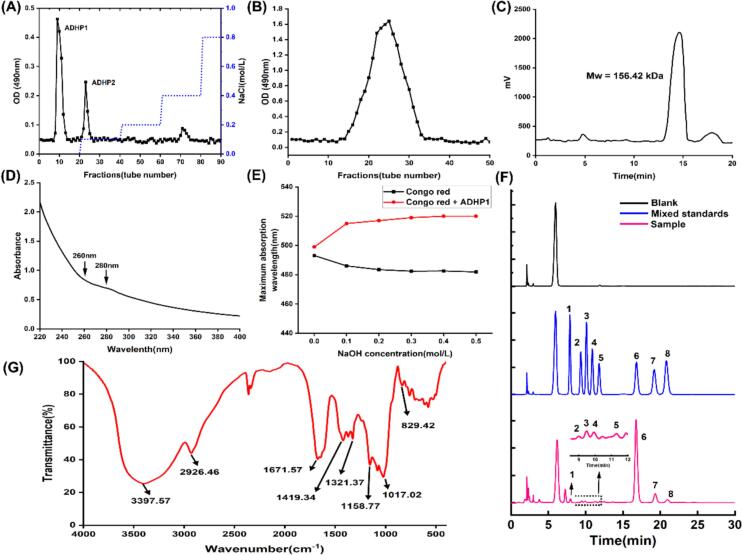
Structural characterization of polysaccharide ADHP1. (A) DEAE-52 ion-exchange chromatography. (B) Sephadex G-200 gel filtration chromatography. (C) HPGPC molecular weight profiling. (D) UV absorption spectrum analysis. (E) Triple helix conformation stability assay. (F) Monosaccharide composition analysis by HPLC: Mixed standard (1: Man; 2: GlcA; 3: Rib; 4: Rha; 5: GalA; 6: Glc; 7: Gal; 8: Ara) vs ADHP1. (G) FTIR spectroscopic.

Chromatographic separation of THD crude polysaccharide via DEAE-52 Cellulose column (Fig. 3A) generated two distinct fractions under stepwise NaCl elution (0, 0.1, 0.2, 0.4 and 0.8 mol/L). ADHP1, eluted with 0 mol/L NaCl, was provisionally classified as a neutral polysaccharide, while ADHP2 (0.1 mol/L NaCl eluate) exhibited acidic characteristics. This study investigated ADHP1. Subjecting ADHP1 to Sephadex G-200 column purification generated a uniform molecular weight profile (Fig. 3B).

#### 3.2.2 The content of protein and uronic acid

UV–vis analysis of ADHP1 revealed no absorption peaks at 260–280 nm (Fig. 3D), confirming the absence of nucleic acids and residual proteins. Quantitative assays corroborated this purity, demonstrating minimal protein contamination (0.73 ± 0.15 %) attributable to progressive depletion during chromatographic purification. Uronic acid content, quantified via the m-hydroxydiphenyl method, measured 1.86 ± 0.21 % – a value consistent with the classification of ADHP1 as a neutral polysaccharide.

#### 3.2.3 Molecular weight of ADHP1

The HPGPC chromatogram of ADHP1 (Fig. 3C) exhibited a single symmetrical peak, consistent with a homogeneous and pure sample. Its molecular weight parameters, calculated using a standard calibration curve, were determined as follows: Mw = 156.42 kDa, Mn = 73.09 kDa, yielding a polydispersity index (Mw/Mn) of 2.14.

#### 3.2.4 Triple helix structure

The triple-helix structure of polysaccharides enables Congo red complexation under alkaline conditions, accompanied by characteristic red-shifting of maximum absorption wavelength. ADHP1 induced a significant red-shift compared to Congo red alone (Fig. 3E), demonstrating successful complex formation. Progressive attenuation of this spectral shift with increasing NaOH concentrations (0–0.5 M) revealed triple-helix dissociation, confirming the structural conformation of ADHP1.

#### 3.2.5 FTIR analysis

The FTIR spectrum of ADHP1 (Fig. 3G) displayed characteristic absorption bands between 500 and 4000 cm^−1^, with prominent signals at 3397.57 cm^−1^ (O–H), 2926.46 cm^−1^ (C–H), 1158.77 cm^−1^ and 1017.02 cm^−1^ (C–O/C–O–C). A distinct absorption band at 1671.57 cm^−1^ was attributed to C=O asymmetric stretching, potentially originating from uronic acid carboxyl groups (–COOH) or aldose aldehyde moieties (–CHO). Notably, the absence of detectable signals near 1735 cm^−1^ ruled out the presence of uronic acid derivatives. Additional weak absorptions at 1419.34 cm^−1^ and 1321.37 cm^−1^ corresponded to C=O symmetric stretching modes.

#### 3.2.6 Monosaccharide composition analysis

Analysis of the monosaccharide composition of ADHP1 by HPLC (Fig. 3**F**) revealed a heteropolysaccharide predominantly composed of glucose (Glc, 85.92 mol%) and galactose (Gal, 8.03 mol%). These results indicate that ADHP1 is a Glc-Gal heteropolysaccharide with Glc as the predominant monosaccharide component, consistent with earlier reports [26].

#### 3.2.7 XRD analysis

**Fig. 4:**
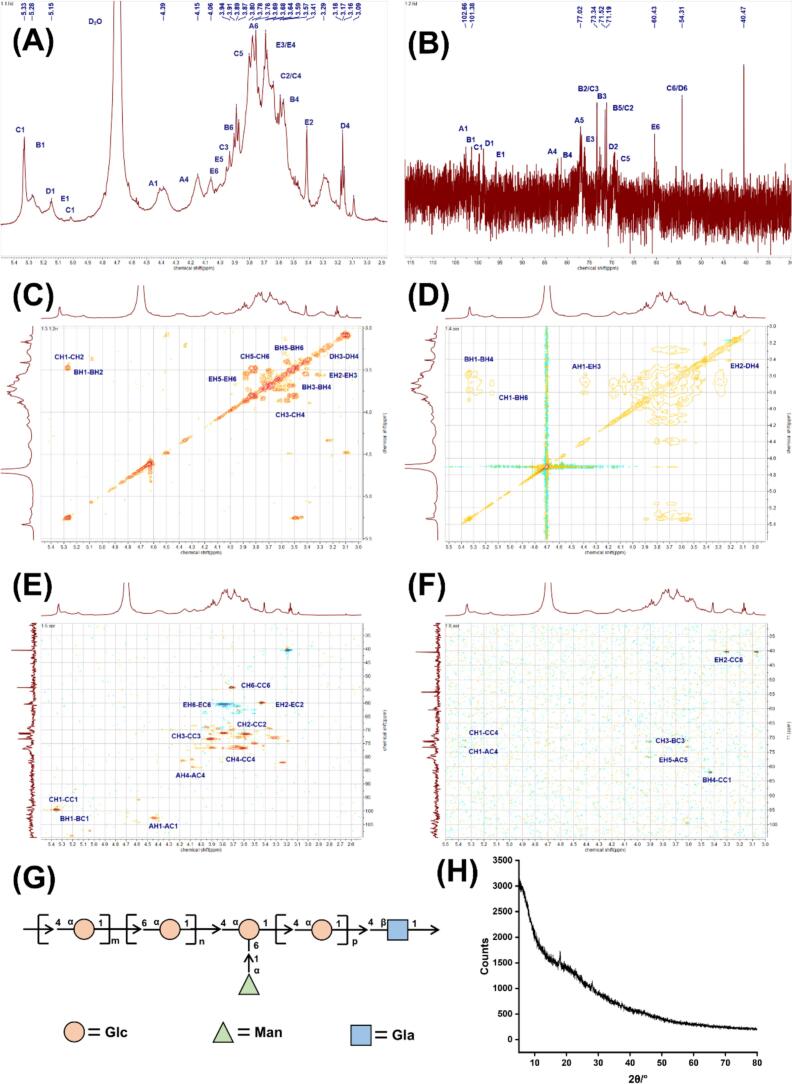
Structural elucidation and crystallographic analysis of polysaccharide ADHP1. (A): ^1^H NMR, (B): ^13^C NMR, (C): ^1^H–^13^C COSY, (D): TOCSY NMR spectra, (E): ^1^H–^1^H HSQC, (F): ^1^H–^13^C HMBC, (G): The structure of ADHP1, (H): XRD analysis of ADHP1.

XRD analysis allows determination of polysaccharide crystallinity, where sharp diffraction peaks typically correspond to crystalline domains and broad peaks to amorphous regions. As shown in Fig. 4 H, the XRD profile of ADHP1 exhibited a characteristic sharp diffraction peak at 18.25°, indicative of crystalline organization. The limited diffraction signals observed at other angles suggest the coexistence of subcrystalline and amorphous structural elements. Quantitative analysis revealed ADHP1 to be predominantly amorphous, with only minor crystalline components。.

#### 3.2.8 Methylation analysis of ADHP1

Methylation analysis was conducted to determine the glycosidic linkage types within ADHP1. The results (Table 5) revealed four glucopyranosyl (Glc*p*) residues: →4)-Glc*p*-(1→ (66.3 %), →6)-Glc*p*-(1→ (31.5 %), →4,6)-Glc*p*-(1→ (0.9 %), →2,3)-Glc*p*-(1→ (0.7 %), along with a →4)-galactopyranosyl (Gal*p*)-(1→ (0.6 %). These data suggest that the backbone of ADHP1 consists of →4)-Glcp-(1→, →6)-Glcp-(1→ and the branches are located at C2, C4, C6 of the →2,3)-Glcp-(1→, →4)-Galp-(1→, →4,6)-Glcp-(1→.

**Table 5:** Methylation analysis of the polysaccharide ADHP1.

Methylated sugar	Linkage pattern	Molar ratio (%)	Mass fragments (*m*/*z*)
2,3,6-Me-D-Glcp	→4)-Glcp-(1→	66.29	43,87,99,101,113,117,129,131,161,173,233
2,3,4-Me-D-Glc	→6)-Glcp-(1→	31.54	43,71,87,99,101,117,129,161,173,189,233
2,3-Me-D-Glcp	→4,6)-Glcp-(1→	0.88	43,85,99,101,117,127,142,159,201,261
4,6-Me-Glcp	→2,3)-Glcp-(1→	0.73	43,87,101,112,128,129,161,202,262
2,3,6-Me-D-Galp	→4)-Galp-(1→	0.56	43,87,99,101,113,117,129,131,161,173,233

#### 3.2.9 NMR analysis of ADHP1

To resolve the complete structural features of ADHP1, comprehensive NMR analyses were performed, including ^1^H NMR, ^13^C NMR, and two-dimensional correlation spectroscopy (2D-COSY, HSQC, HMBC). As shown in Fig. 4A, characteristic anomeric proton signals (C1, *δ* 4.0–5.5 ppm) exhibited well-resolved splitting patterns. In contrast, non-anomeric protons (C2-C6, *δ* 3.0–4.0 ppm) displayed extensive signal overlap in the ^1^H NMR spectrum, necessitating 2D spectral analysis for unambiguous assignment. This resonance pattern confirms the presence of both α- and β-linked glycosyl units with complex branching topology. Therefore, Fig. 4(A) was mainly used to distinguish the conformations of glycosidic bonds in the polysaccharide structure and to further confirm the analytical results of Fig. 4(B). NMR identified characteristic α-glycosidic bond configurations in ADHP1, consistent with the diagnostic downfield proton shifts (>5 ppm) observed for α-linked glycans.

The ^13^C NMR spectrum of ADHP1 (Fig. 4B) revealed distinct carbon resonance regions: anomeric carbons (*δ* 95–110), C2–C6 skeletal carbons (*δ* 55–85), and galactopyranosyl C-1 signals (*δ* 103–106). The anomeric region (*δ* 95–110) displayed characteristic α- and β-glycosidic configurations, with β-linked residues typically resonating above *δ* 100. The clustered Galp C-1 signals (*δ* 103–106) suggest multiple substitution patterns of terminal galactose units, while the broad C2–C6 resonance implies conformational flexibility in the glucopyranosyl backbone.

The glycosidic linkage architecture of ADHP1 was resolved through integrated analysis of a multidimensional NMR dataset comprising ^1^H, ^13^C, and two-dimensional correlation spectra [27]. The following assigned signals were obtained: *δ*102.66/5.33 ppm, *δ*101.38/5.15 ppm, *δ*77.02/3.69 ppm, *δ*71.19/3.57 ppm, *δ*60.43/3.76 ppm, *δ*54.31/3.68 ppm and *δ*40.47/3.17 ppm. Resonances at *δ* 102.66/5.33 ppm correspond to glucose in α-configuration, while signals at *δ*102.66/5.33 and *δ*101.38/5.15 ppm are typical for substituted C1/H1 and C4/H4 of (1 → 4)-α-Glcp and (1 → 6)-α-Glcp. It was concluded that → 4)-Glcp-(1 → and → 6)-Glcp-(1 → was the main chain of ADHP1. The absence of downfield ^13^C NMR signals in the *δ* 170–180 region confirmed that ADHP1 does not contain uronic acid. Chemical shifts (*δ*, ppm) for all identified sugar residues are provided in Table 6. Combined with methylation product analysis, the NMR data establish that ADHP1′s structure comprises the structural fragment depicted in Fig. 4G.

**Table 6:** ^1^H and ^13^C NMR chemical shifts for ADHP1.

Sugar residues	Chemical shifts (*δ*, ppm)				
	H1/C1	H2/C2	H3/C3	H4/C4	H5/C5
→4)-Glcp-(1→	5.33/99.67	3.57/71.49	3.90/73.33	3.57/76.71	3.77/71.14
→6)-Glcp-(1→	4.45/101.91	3.24/72.07	3.44/74.51	3.57/73.73	3.61/73.80
→4,6)-Glcp-(1→	4.90/97.95	3.55/71.51	3.73/72.71	3.58/73.37	3.89/71.21
→2,3)-Glcp-(1→	5.37/98.4	3.59/73.2	3.70/72.9	3.51/70.7	3.80/73.1
→4)-Galp-(1→	5.16/93.52	3.69/69.26	3.83/69.87	4.43/79.94	4.27/71.48

### 3.3 In vivo antipyretic activity

**Fig. 5:**
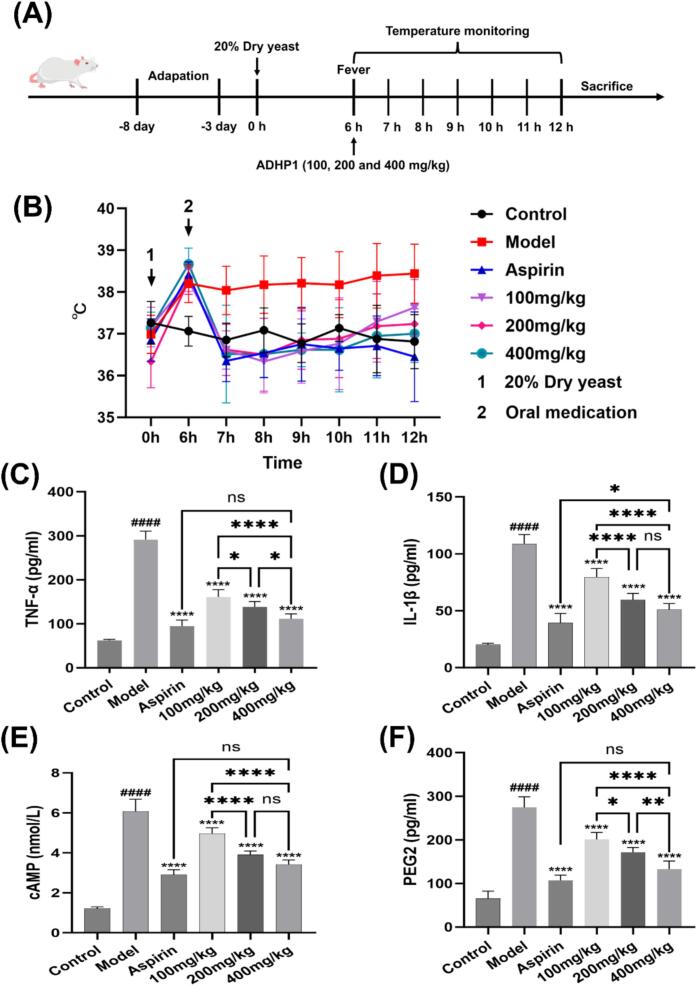
Antipyretic efficacy of polysaccharide ADHP1 in yeast-induced fever model. (A) Experimental workflow schematic. (B) Anal temperature dynamics post-induction. (C-F) Serum biomarker modulation: (C) TNF-α, (D) IL-1β, (E) cAMP, (F) PGE2 levels. Multi-way ANOVA was used to calculate significant difference, Control vs Model ####p < 0.0001, ##p < 0.01; Each administration group vs Model *p < 0.05, **p < 0.01, ***p < 0.001, ****p < 0.0001.

To assess the antipyretic activity of ADHP1, we employed a mice fever model induced by 20 % dry yeast suspension, with experimental parameters detailed in Fig. 5(A). As shown in Fig. 5(B), 20 % dry yeast suspension (10 ml/kg) elicited a pronounced temperature change in contrast to control group (ΔT ≈ 1.5 °C). Aspirin and ADHP1 was administrated only once at 6 h after 20 % dry yeast suspension subcutaneous injection. The data showed that aspirin exhibited a potent and lasting antipyretic effect after intragastric administration. After administration of ADHP1, although the temperature curves of three ADHP1 groups were all below the model group during 6 h detection course, only 400 and 200 mg/kg of ADHP1 showed a significant antipyretic effect at the time point of 6 h after 20 % dry yeast challenge. Administration of ADHP1 (100 mg/kg) elicited a progressive increase in mice anal temperature commencing 2 h post-dosing, reaching comparable levels to the model group with no statistically significant divergence at the 6-h timepoint.

During febrile responses, exogenous pyrogens initiate immune activation by stimulating macrophages, lymphocytes, and monocytes, thereby inducing endogenous pyrogen production. These mediators – including key inflammatory factors such as TNF-α, IL-1β, and PGE_2_ – subsequently mediate thermoregulatory center modulation through direct and indirect pathways [23]. Therefore, we examined the effects of ADHP1 on TNF-α, IL-1β, cAMP and PGE_2_ levels in vivo (Fig. 5C–F). Administration of 20 % dry yeast suspension triggered robust elevation of key inflammatory mediators (TNF-α, IL-1β, cAMP, PGE2) relative to control group. Both aspirin and ADHP1 demonstrated significant reductions in these pro-inflammatory factors compared with model group levels, showing comparable therapeutic efficacy. Following the same trend as temperature, the effect of ADHP1 was dose-dependent, but less effective than that of aspirin. Taken together, these results indicated that ADHP1 exerted a confirmed but mild antipyretic effect.

### 3.4 Identification of DEGs in datasets

**Fig. 6:**
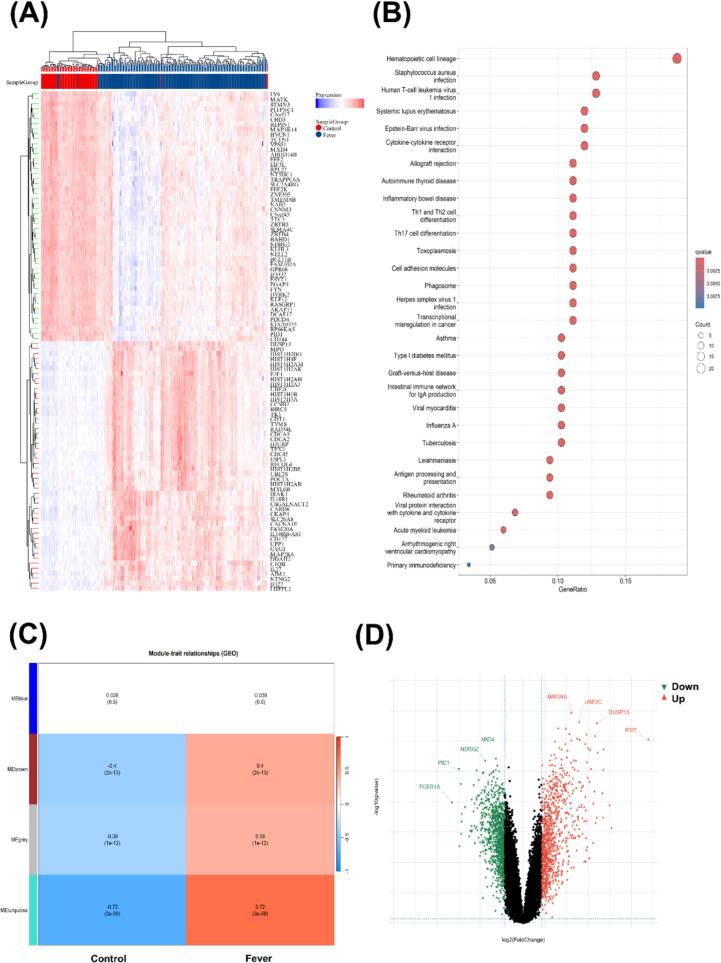
(A): Heat map of differential analysis between fever and control group. (B): KEGG enrichment bubble chart. (C): The connection between modules and traits. (D): Hot map of DEGs.

Control and fever groups were assigned datasets GSE185576 and GSE101702, respectively. Differential expression analysis using Student's *t*-test identified 1,037 downregulated and 856 upregulated genes (Fig. 6D). Among them, MAP2K6, UBE2C, DUSP13 and IFI27 were the most significantly up-regulated genes, while MXD4, NDRG2, PID1 and FCR1A were the most significantly down-regulated genes. WGCNA of GSE185576 and GSE101702 identified four distinct modules (Fig. 6C). The MEturquoise module demonstrated the strongest correlation with febrile responses (r = 0.72, P = 3e−50), containing 222 hub genes central to the observed thermoregulatory phenotype. KEGG pathway enrichment analysis indicated these genes were primarily involved in viral infection, inflammation, and immune system-related diseases (Fig. 6B). A total of 5 key genes related to fever were screened by PPI in MEturquiose, including ELANE, CTSG, LTF, MPO and PRTN3 [[28], [29], [30], [31], [32]].

### 3.5 Molecular docking

**Fig. 7:**
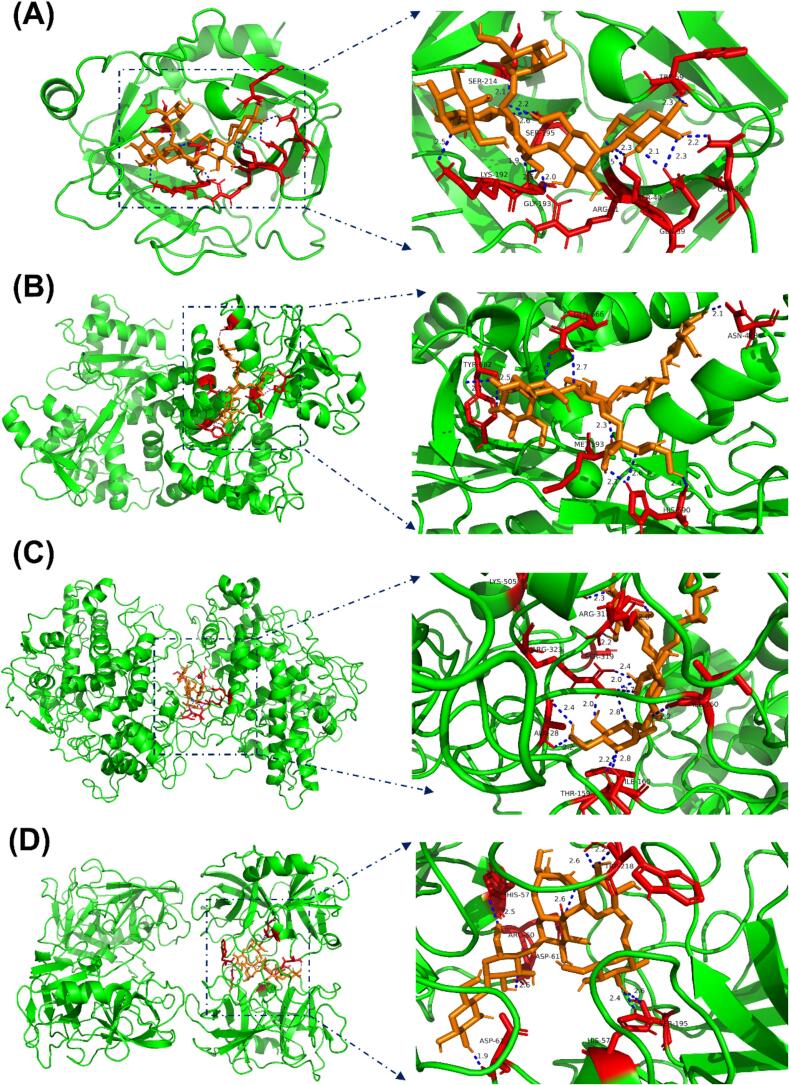
Molecular interaction profiling of ADHP1. (A) CTSG binding interface. (B) LTF ligand coordination. (C) MPO active-site docking. (D) PRTN3 catalytic pocket interaction.

Pymol software was used to dock the significantly changed differential genes with ADHP1 (Fig. 7). The binding energies were calculated using scoring functions. The binding energies were −6.3 kcal/mol for ELANE, −7.5 kcal/mol for CTSG, −7.5 kcal/mol for LTF, −9.4 kcal/mol for MPO and −9.0 kcal/mol for PRTN3. The number of hydrogen bonds between ADHP1 and ELANE, CTSG, LTF, MPO, and PRTN3 was 4, 13, 10, 13, and 8, respectively. Among them, ADHP1 has strong binding with CTSG, LTF, MPO and PRTN3 (binding energies <−7 kcal/mol) [33]. These findings suggest that ADHP1 could be potential activator of CTSG, LTF, MPO and PRTN3 to exert antipyretic activity.

## 4 Discussion

Herbal compounds are promising candidates for drug development owing to their unique polysaccharides, which are not endogenously produced in humans. Optimization of polysaccharide extraction processes can be achieved through multiple approaches, with orthogonal designs and Box-Behnken designs emerging as the most prevalent methodologies [[34], [35], [36]]. Despite growing interest in extraction optimization, systematic evaluation of model suitability remains underexplored. The Box-Behnken design typically characterizes input–output relationships through constructing response surface models, with specific implementation protocols dictated by experimental constraints. However, this methodology demonstrates marked reduction in model fidelity with increasing variable dimensionality, manifesting as statistical insignificance of regression parameters, diminished predictive capacity, and ultimately compromised model validity [37].

Artificial neural networks demonstrate superior capacity in processing complex datasets through continuous self-optimization via iterative weight and threshold adjustments, progressively enhancing predictive accuracy. This computational plasticity is particularly advantageous for addressing nonlinear phenomena in biochemical systems. Moreover, these architectures exhibit dynamic architectural reconfiguration and parameter space adaptation, enabling robust responsiveness to evolving experimental conditions and environmental variables [38].

In this experiment, comparative analysis of three models revealed distinct response patterns in polysaccharide extraction modeling. Response surface analysis demonstrated monotonic input–output relationships in Box-Behnken designs, whereas the GA-ACO-BP hybrid model exhibited nonlinear interdependencies through multidimensional feature mapping. Notably, neural implementations present inherent constraints including data-intensive training requirements that limit effectiveness in small-sample regimes. Parametric complexity frequently precipitates overfitting, thereby eroding model generalizability. Furthermore, suboptimal initialization protocols may entrap optimization in local minima during parameter space exploration. These findings advocate hybrid modeling frameworks integrating multiple optimization paradigms to enhance predictive robustness in phytochemical process engineering [39].

The pharmacological efficacy of polysaccharides exhibits pronounced structure–activity correlations governed by their supramolecular architectures. Critical structural determinants include molecular weight, glycosidic linkage patterns, backbone stereochemistry, branching topology, and three-dimensional conformations, which collectively mediate biointerfacial recognition processes [40,41]. Elucidating structure–activity correlations in polysaccharides remains challenging; however, emerging evidence suggests potential relationships may be inferred. Structural analysis using FT-IR spectroscopy identified ADHP1 as an α-glucan with a molecular weight of 156.42 kDa. Structural elucidation of ADHP1 remains challenging owing to its water insolubility, with limited studies addressing its primary architecture. NMR analysis has revealed (1 → 4)-α-Glcp glycosidic linkages in the main chain, while side chain configurations remain uncharacterized. The poor aqueous solubility of ADHP1 in D_2_O induces elevated solution viscosity, compromising the broad applicability of NMR for polysaccharide structural characterization. Methylation analysis, a cornerstone technique in carbohydrate chemistry, revealed →4)-Glcp-(1→ and →6)-Glcp-(1→ linkages with branching at C-6, C-2, and C-4 positions.

Congo red binding analysis revealed a triple-helical conformation in ADHP1. While no direct correlation has been established between polysaccharide helical configurations and febrile responses, this structural motif demonstrates significant attenuation of pro-inflammatory cytokine levels in murine fever models [42]. Triple-helical polysaccharides demonstrate stereoselective engagement with immune cell surface receptors, mediating targeted molecular recognition events [43]. Polysaccharides with elevated molecular weight (Mw) display enhanced biofunctional profiles, attributed to their capacity for multivalent binding topologies with cellular receptors. This structure–activity paradigm underpins observed potent immunomodulatory responses, particularly manifested in macrophage activation and cytokine induction [44]. ADHP1 (molecular weight 156.42 kDa) has a higher molecular weight and stronger immunostimulatory activity.

Integrative bioinformatics interrogation combining GEO dataset mining with PPI networks and WGCNA identified five hub genes mechanistically linked to febrile pathogenesis. Multi-omics validation through KEGG pathway and GO enrichment analyses confirmed these targets as nodal regulators in immune-dysregulation cascades [45]. Molecular docking results suggested that CTSG, LTF, MPO and PRTN3 may play an important role in ADHP1 treatment of fever [[28], [29], [30], [31], [32]]. These findings position ADHP1 as a promising drug compound candidate for febrile disorders, while comprehensive mechanistic delineation remains imperative to decipher its pharmacodynamic interplay within immunometabolic cascades.

## 5 Conclusion

Process optimization through the integration of response surface methodology and artificial neural network modeling achieved an optimal THD polysaccharide yield of 19.11 ± 0.33 % under defined parameters: liquid-to-solid ratio (30 mL/g), ultrasonic power (260 W), extraction temperature (100 °C), and duration (90 min). The purified ADHP1 fraction represents a neutral α-glucan (Mw 156.42 kDa) featuring a triple-helical architecture with →4)-Glcp-(1→ and →6)-Glcp-(1→ backbone linkages and C-2/C-4/C-6 branching patterns. In mice fever models, ADHP1 demonstrated marked antipyretic efficacy through dual suppression of pro-inflammatory cytokines (IL-1β, TNF-α) and prostaglandin biosynthesis. Systems pharmacology interrogation integrating GEO datasets, PPI networks, and molecular docking identified CTSG, LTF, MPO, and PRTN3 as pivotal targets mediating ADHP1′s immunothermal regulation. These results show the potential of ADHP1 as a promising antipyretic and provide new insights into the rational utilization of THD.

## CRediT authorship contribution statement

**Zhongpeng Ding:** Writing – review & editing, Writing – original draft, Methodology, Investigation. **Ningchen Zhang:** Writing – review & editing, Writing – original draft, Methodology, Investigation. **Meihong Ding:** Writing – review & editing, Writing – original draft, Methodology, Investigation. **Shiyi Zhang:** Visualization, Data curation. **Xiaowen Yao:** Visualization, Data curation. **Yu Xia:** Visualization, Data curation. **Senlin Shi:** Writing – review & editing, Supervision, Project administration.

## Declaration of competing interest

The authors declare that they have no known competing financial interests or personal relationships that could have appeared to influence the work reported in this paper.
